# Clinical evaluation on automatic segmentation results of convolutional neural networks in rectal cancer radiotherapy

**DOI:** 10.3389/fonc.2023.1158315

**Published:** 2023-09-05

**Authors:** Jing Li, Ying Song, Yongchang Wu, Lan Liang, Guangjun Li, Sen Bai

**Affiliations:** ^1^Radiotherapy Physics & Technology Center, Cancer Center, West China Hospital, Sichuan University, Chengdu, China; ^2^Machine Intelligence Laboratory, College of Computer Science, Chengdu, China

**Keywords:** automatic segmentation, deep learning, rectal neoplasms, radiotherapy, CNN

## Abstract

**Purpose:**

Image segmentation can be time-consuming and lacks consistency between different oncologists, which is essential in conformal radiotherapy techniques. We aimed to evaluate automatic delineation results generated by convolutional neural networks (CNNs) from geometry and dosimetry perspectives and explore the reliability of these segmentation tools in rectal cancer.

**Methods:**

Forty-seven rectal cancer cases treated from February 2018 to April 2019 were randomly collected retrospectively in our cancer center. The oncologists delineated regions of interest (ROIs) on planning CT images as the ground truth, including clinical target volume (CTV), bladder, small intestine, and femoral heads. The corresponding automatic segmentation results were generated by DeepLabv3+ and ResUNet, and we also used Atlas-Based Autosegmentation (ABAS) software for comparison. The geometry evaluation was carried out using the volumetric Dice similarity coefficient (DSC) and surface DSC, and critical dose parameters were assessed based on replanning optimized by clinically approved or automatically generated CTVs and organs at risk (OARs), *i.e.*, the Plan_ref_ and Plan_test_. Pearson test was used to explore the correlation between geometric metrics and dose parameters.

**Results:**

In geometric evaluation, DeepLabv3+ performed better in DCS metrics for the CTV (volumetric DSC, mean = 0.96, P< 0.01; surface DSC, mean = 0.78, P< 0.01) and small intestine (volumetric DSC, mean = 0.91, P< 0.01; surface DSC, mean = 0.62, P< 0.01), ResUNet had advantages in volumetric DSC of the bladder (mean = 0.97, P< 0.05). For critical dose parameters analysis between Plan_ref_ and Plan_test_, there was a significant difference for target volumes (P< 0.01), and no significant difference was found for the ResUNet-generated small intestine (P > 0.05). For the correlation test, a negative correlation was found between DSC metrics (volumetric, surface DSC) and dosimetric parameters (δD95, δD95, HI, CI) for target volumes (P< 0.05), and no significant correlation was found for most tests of OARs (P > 0.05).

**Conclusions:**

CNNs show remarkable repeatability and time-saving in automatic segmentation, and their accuracy also has a certain potential in clinical practice. Meanwhile, clinical aspects, such as dose distribution, may need to be considered when comparing the performance of auto-segmentation methods.

## 1 Introduction

Preoperative radiotherapy is currently considered to be the standard treatment for locally advanced rectal cancer and has been proven to reduce local recurrence (1–3). With the development of radiotherapy technology, such as intensity modulated radiotherapy (IMRT) and volumetric modulated arc therapy (VMAT), the target volume can receive a highly conformal dose distribution (4). In addition, it has been proven that IMRT and VMAT are dosimetrically superior to other conformal techniques in protecting organs at risk (OARs) in rectal cancer (5). Thus, the accurate delineation of the clinical target volume (CTV) and OARs is crucial for treatment planning in rectal cancer.

Interobserver differences occur during manual delineation, which depend on oncologists’ clinical experience, resulting in significant changes in dose distributions (6). Multiple studies have applied deep learning methods to automatic segmentation to solve the problem of time consumption and the lack of consistency in manual contouring (7–9). Based on the planning computed tomography (pCT) images, the oncologists’ delineated regions of interest (ROIs) as a training set. These structures are imported into deep learning models with CT images, and their corresponding features are extracted to train models according to the framework characteristics of different models.

The accuracy of automatic segmentation requires clinical evaluation. Objective evaluation metrics such as the volumetric Dice similarity coefficient (volumetric DSC) and Hausdorff distance (HD) are widely used, and some studies have carried out dosimetry assessments (10–12). However, clinical evaluation of the quality of deep learning delineation has limitations (13). Considering the different accuracy requirements of CTVs and OARs in clinical practice, it is necessary to combine their clinical importance and tolerant errors and carry out a comprehensive evaluation from the perspectives of geometry and dosimetry.

We carried out a retrospective study of radiotherapy patients with rectal cancer. CTV and OARs were segmented manually as the ground truth (GT), two convolutional neural networks we have trained—DeepLabv3+ and ResUNet—were used for automatic delineation (13), and a common method Atlas-based Auto segmentation (ABAS) was used as a comparison. Our research aimed to explore the clinical impact of auto-segmentation results from a dosimetric perspective.

## 2 Materials and methods

### 2.1 Patient data

The retrospective study was approved by the ethics committee of West China Hospital in 2019, with no extra health risks and no need for patient consent. Rectal cancer patients from February 2018 to April 2019 at West China Hospital were chosen randomly and metastases in advanced patients were ignored. Each patient was immobilized in a supine position with arms over the head using a radiotherapy thermoplastic mold, and this position was applied during simulation and treatment. The contrast-enhanced CT images (tube voltage, 120 kVp; matrix size, 512 × 512; voxel resolution, 0.9 × 0.9 × 3.0 mm in left-right, antero-posterior and cranio-caudal directions) were acquired as patient pCT on the same CT scanner (SOMATOM Definition AS, Siemens Healthcare).

Based on the pCT, a radiation oncologist manually segmented the CTV and OARs of rectal cancer by referring to Radiation Therapy Oncology Group consensus guidelines. Then, the structures were modified and approved by a senior expert physician and labelled as ground truth, including CTV_GT_, bladder_GT_, small intestine_GT_, left femoral head_GT_ and right femoral head_GT_.

### 2.2 Automatic segmentation

**Figure 1:**
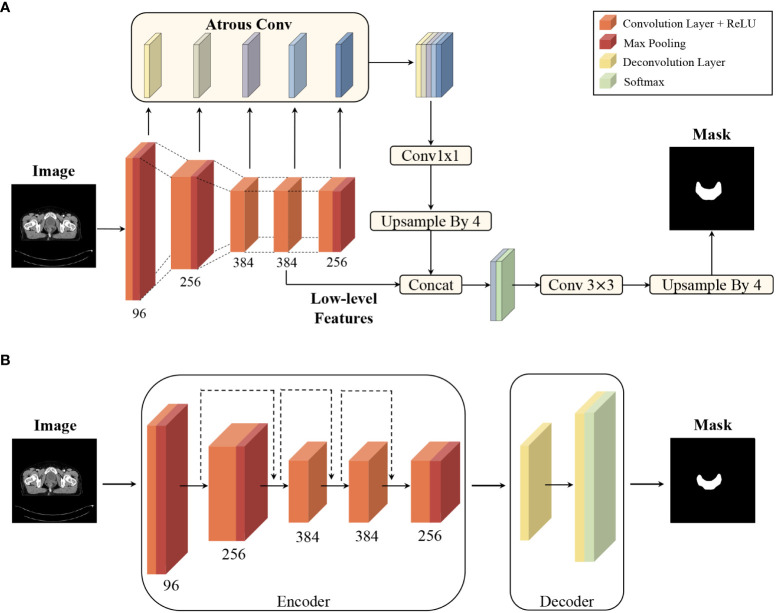
Convolutional neural network architecture diagram: **(A)** architecture of DeepLabv3+ for segmentation, and **(B)** architecture of ResUNet for segmentation. The two networks had been implemented using data sets from 199 patients (training set with 98 cases, validation set with 38 cases, and test set with 63 cases) in previous study (13).

DeepLabv3+ and ResUNet, two typical CNNs, were used for automating delineation. DeepLabv3+ employs an atrous spatial pyramid pooling module and concat aggregation for the extraction and integration of high-level features, and ResUNet has shortcut connections for each level of features (13). The pCT images of all patients were imported into the models to obtain the mask of each structure on every CT slice. The two-dimensional masks were then converted to a three-dimensional structure in DICOM format, imported to the treatment planning system (TPS), and labelled as ROI_DeepLabv3+_ and ROI_ResUNet_, respectively. The network models were uploaded onto github (https://github.com/hujunjiescu/DeepRadiology_rectum), and the architecture diagram was shown in **Figure 1**.

CNN models had the same training settings. The contouring tasks were worked out based on the Pytorch deep learning framework using Python. The experiments were performed on a Linux operating system workstation with the CPU Intel Xeon E5-2620 v3@ 2.4GHz, GPU NVIDIA Tesla K40 Xp, and 64 GB RAM. The loss function for the optimization was the weighted cross-entropy, which was defined as:


$$J\;=\;-\frac{1}{N}\sum_{i=1}^{N}\sum_{c=1}^{C}w^{c}y_{i}^{c}\ln\left(a_{i}^{c}\right)$$

where N, C, w, y, and a denoted the batch size, number of classes, weight factor, ground truth sets, and prediction sets, respectively. The batch size N was set at 10, the weight factor w at 2, and the total training epoch *T* at 100. The stochastic gradient descent method was used to optimize the network with the initial learning rate set as 0.01, which was multiplied by ${\left(1-\frac{t}{T}\right)}^{0.9}$ for the epoch *t*. The segmentation results were rewritten into DICOM RT structure (RS) files based on their original spatial resolutions.

The ABAS worked on CT datasets using a multi-patient atlas. We randomly selected 5 atlas patients from the CNN training set, then their pCT images and manual contoured structures were imported to ABAS software (Version 2.01.00, Elekta CMS, Inc.). The Simultaneous Truth And Performance Level Evaluation (STAPLE) algorithm was used to fuse the multiple single-subject atlas auto-segmentation sets into one multi-subject auto-segmentation set (6).

### 2.3 Treatment plans

To evaluate the clinical dosimetry value of the two automatic delineation methods, a two-round optimization protocol was performed using TPS (Raystation, version 4.7.5, Raystation Laboratories, Stockholm, Sweden): 1) The corresponding PTV was obtained based on CTV expanded with a three-dimensional margin of 5 mm; 2) The dose prescription was set to 50.4Gy/28 fraction to the PTV; 3) Two full arcs, one from 181 to 180° clockwise and the other from 180 to 181° counterclockwise, were designed using the VMAT technique and 6 MV photons; 4) The initial optimization parameters applied to the first round VMAT planning were shown in **Table 1**, and in the second round, the weight of Parameter_4_ was set to 100, and the weight of Max EUD objectives was set to 0.01.

**Table 1:** Initial planning objective set for plan optimization.

Parameter (P_i_)	ROI	Description	Weight
P_1_	PTV	Min Dose 50.4Gy	60
P_2_	PTV	Max Dose 52.42Gy	90
P_3_	PTV	Min DVH 50.9Gy to 95% volume	100
P_4_	PTV	Uniform Dose 51.21Gy	0.2
P_5_	Bladder Avoid	Max DVH 40Gy to 52% volume	20
P_6_	Bladder Avoid	Max EUD 28Gy, Parameter *α* 1	1
P_7_	Small intestine Avoid	Max DVH 30Gy to 30% volume	20
P_8_	Small intestine Avoid	Max EUD 20Gy, Parameter *α* 1	1
P_9_	Femoral Head Right	Max DVH 40Gy to 5% volume	20
P_10_	Femoral Head Right	Max EUD 1500, Parameter *α* 1	1
P_11_	Femoral Head Left	Max DVH 40Gy to 5% volume	20
P_12_	Femoral Head Left	Max EUD, Parameter *α* 1	1
P_13_	External	Dose Fall-Off [H]50.4Gy [L]25.2Gy, [D]2.8 cm	15

Taking the difficulty of CTV delineation into consideration, we divided the results of the three auto-segmentation methods into CTV and OAR groups and introduced them as optimization objectives separately to obtain Plan_test_: 1) the plan optimized using CTV_DeepLabv3+_ and OAR_GT_ labelled Plan1; 2) the plan optimized using CTV_ResUNet_ and OAR_GT_ labelled Plan2; 3) the plan optimized using CTV_ABAS_ and OAR_GT_ labelled Plan3; 4) the plan optimized using CTV_GT_ and OAR_DeepLabv3+_ labelled Plan4; 5) the plan optimized using CTV_GT_ and OAR_ResUNet_ labelled Plan5; and 6) the plan optimized using CTV_GT_ and OAR_ABAS_ labelled Plan6. In addition, we obtained a Plan_ref_ optimized using ROI_GT_, calculated characteristic dose parameters of ROI_GT_ in all plans, and compared parameters extracted from Plan_test_ with those from the Plan_ref_ respectively.

### 2.4 Evaluation metrics and statistical analysis

In terms of geometry, two DSCs were used to evaluate quantitatively, which were calculated on the overlap of the ROI structures. The ROIs were converted from RS files to thresholding masks, and the masks were divided into slices corresponding to the CT images. The mathematical operations were carried out based on all mask slices of a certain structure and the average was obtained as DSC value.

The volume similarity was usually evaluated by volumetric DSC, calculated using:


$$\text{Volumetric DSC = }\frac{2\left(V_{1}\cap^{\;}V_{2}\right)}{V_{1}\cup^{\;}V_{2}}$$

where V_1_ were the ROIs of ground truth set and V_2_ were the corresponding auto-segmentation structures. Volumetric DSC varies between 0 (no overlap) and 1 (complete overlap), which indicates the degree of overlap between ROI_GT_ and auto-segmentation results.

**Figure 2:**
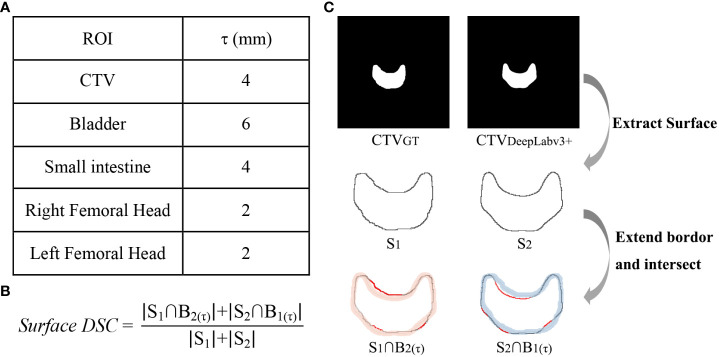
Calculation method of surface DSC. **(A)** acceptable tolerance τ value, **(B)** surface DSC formula, **(C)** the calculation process taken CTV as an example, in which the red lines in ${\text{S}}_{1}\cap {\text{B}}_{2(\tau );}$ and ${\text{S}}_{2}\cap {\text{B}}_{1(\tau );}$ were the part that exceeds the tolerance.

To characterize the proportion of the contour edges that need to be redrawn, the surface DSC was applied to assess the agreement between just the surface of two contours (14). The surface DSC represents the proportion of units with acceptable distance, and the acceptable tolerance τ was defined as the 95th percentile of the distance difference contoured by two oncologists for each structure. As shown in **Figure 2**, the masks of two ROIs to be compared were used to extract the contour surface and labelled S, then the surface border was expanded by τ both inside and outside to get B(τ). Currently, the parts of one structure’s S that does not coincide with the other structure’s B(τ) were regarded as exceeding the tolerance.

In addition, the clinical practicability of contours delineated automatically is evaluated by the accuracy of the dose distribution in plan design. The characteristic dosimetry parameters of ROI_GT_ in every plan were extracted for comparison. D2 (Dn representing the absolute dose of n% volume) and D98 were extracted to signify hot spots and cold spots for all structures, respectively (4). V50.4 for CTV indicated whether CTV received enough dose, while the conformal index ( $CI=\frac{{TV}_{RI}}{TV}\times \frac{{TV}_{RI}}{V_{RI}}$ , where TV is target volume, TV_RI_ is the target volume covered by the 95% prescription dose, and V_RI_ is the volume of the 95% prescription dose) and homogeneity index ( $HI=\frac{D_{2}{-D}_{98}}{D_{50}}$ ) were used to evaluate PTV (15, 16). Moreover, relative dose parameters Vn (volume percentage receiving radiation ≥n Gy) related to acute or late toxicity of OARs were obtained for all plans, including V30, V40, V50 of the bladder, V15, V45, V50 of the small intestine, and V40, V45 of the femoral head (17–20).

The collected data were analyzed using SPSS Statistics software (version 26.0, SPSS Inc., Chicago, IL, United States). Normality tests were performed on all datasets of geometric and dosimetric parameters. Paired samples *t* tests or Wilcoxon signed rank tests were chosen for group comparison with statistical significance set at P value< 0.05 (2-tailed). To make a more intuitive comparison, we calculated the absolute difference between the dose parameters extracted from the Plan_ref_ and the Plan_test_, denoted as D_Abs_, and carried out a statistical description. In particular, Vn, HI, and CI were relative values and directly subtracted, while Dn were absolute values and converted to normalized dose difference ( $\delta Dn\;=\;\frac{\left|{\text{D}}_{{\text{n}}_{\text{test plan}}}{\text{-D}}_{{\text{n}}_{\text{reference plan}}}\right|}{\text{prescription dose}}\times 100\%$ ) (21, 22). In addition, we used the Pearson test to check whether volumetric and surface DSC were correlated and explore whether the geometric metrics of a structure were correlated with its corresponding dose parameters, and the degree of linear correlation.

## 3 Results

Forty-seven rectal cancer patients were included in the study. The median age was 54 years, with a interquartile range (IQR) of 13.97, and other characteristics are shown in **Table 2**. For patients diagnosed with stage IV disease, the study ignored metastases in the training and evaluation. In 5 cases, the structures were not successfully generated from the ABAS software.

**Table 2:** Characteristics of 47 patients.

Characteristic	Value
Sex	
Male	23 (49%)
Female	24 (51%)
Age	
Median (range)	54 (28-83)
Cancer classification	
I	4 (9%)
II	5 (11%)
III	32 (68%)
IV	6 (13%)

**Figure 3:**
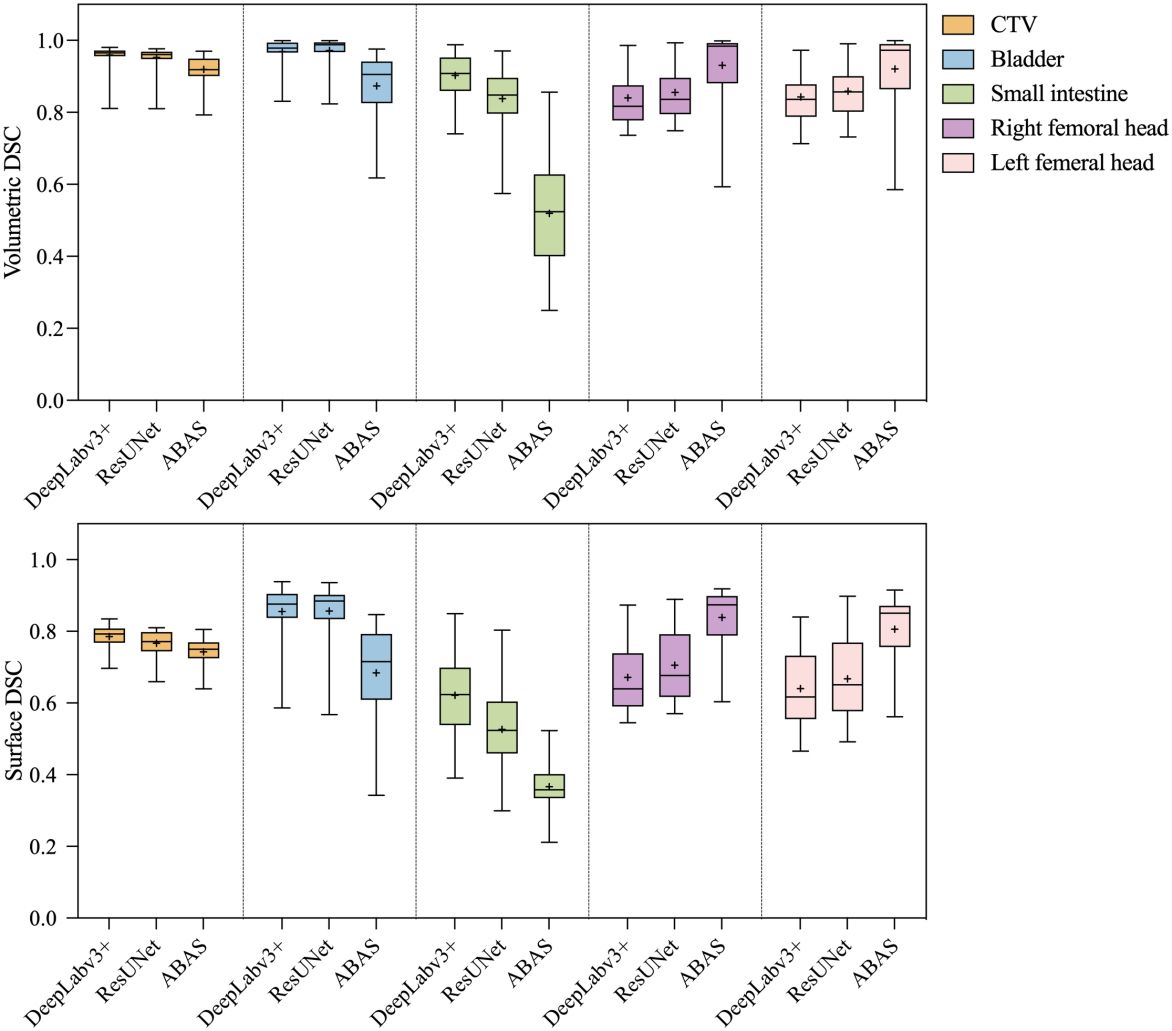
Statistical analysis results of volumetric and surface DSC. The paired- sample tests were performed between the three auto-segmentation results at a significance level of 0.05(2-tailed), and the missing data in the ABAS dataset (*n*=42) were replaced with the mean.

**Figure 4:**
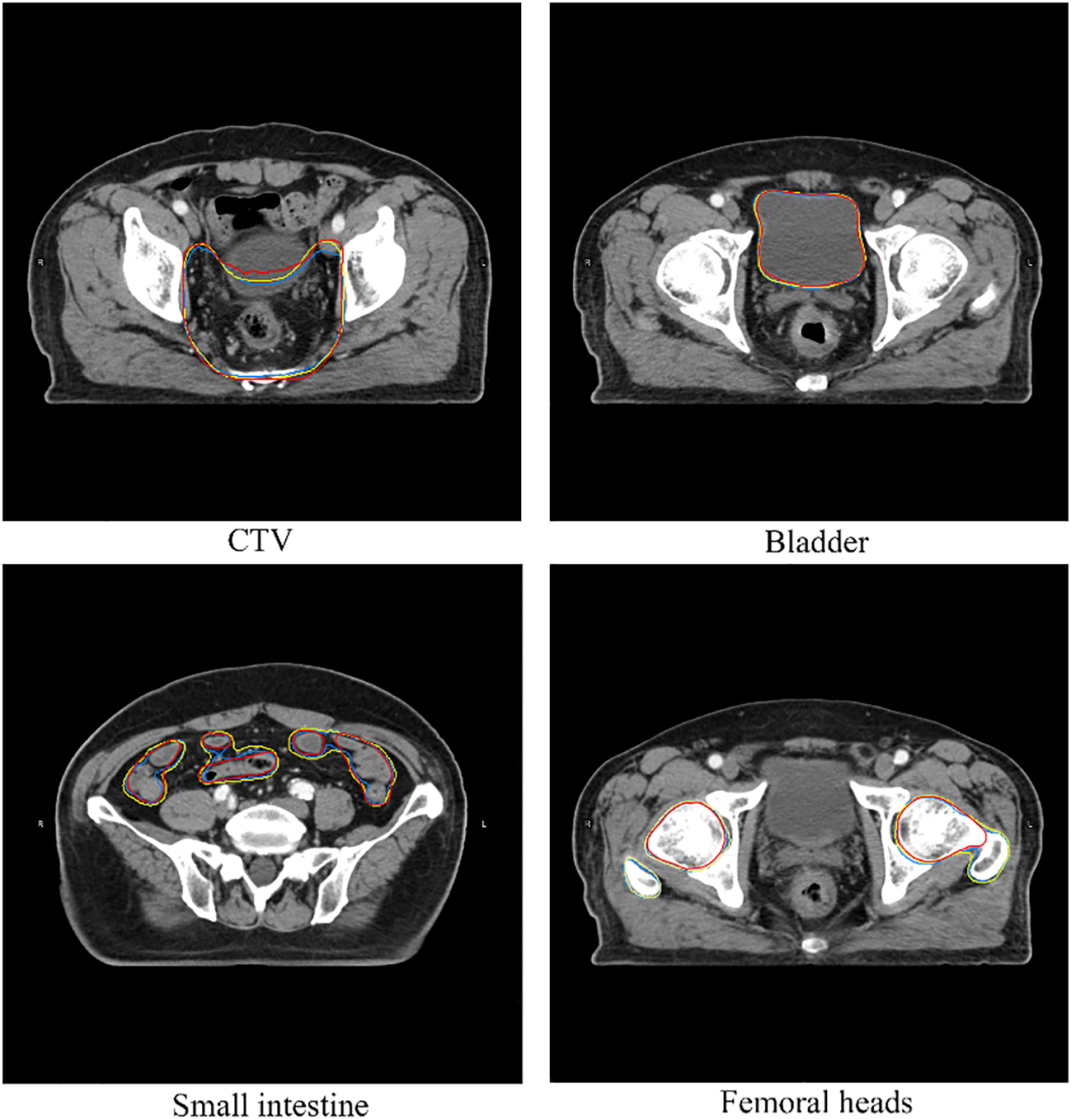
A case of structures comparison. The red line represents ground truth, the yellow line represents automatic segmentation of DeepLabv3+, the blue line represents automatic segmentation of ResUNet.

The statistical analysis results of volumetric and surface DSC are shown in **Figure 3**. In general, the volumetric and surface DSC of the three automatic segmentation results were significantly different, except for the surface DSC of the bladder structure delineated by DeepLabv3+ and ResUNet (P = 0.78). For CTV, DeepLabv3+ showed the best performance on volumetric DSC (mean = 0.96, P< 0.01) and surface DSC (mean = 0.96, P< 0.01). The DSCs of CNNs automatic contouring bladder were significantly higher than those of ABAS (P< 0.01), although there was no significant difference in surface DSC between the two CNNs, the mean value of ResUNet was slightly higher than that of DeepLabv3+ (Bladder_DeepLabv3+_ *vs.* Bladder_ResUNet_, 0.82 *vs*. 0.85, P = 0.78). In the delineation of the small intestine, DeepLabv3+ showed significant advantages, whose mean DSCs (volumetric DSC, 0.91; surface DSC, 0.62) were greater than those of the other two groups (P< 0.01), as well as a lower standard deviation (volumetric DSC, 0.05; surface DSC, 0.10). For the segmentation of the right and left femoral head, ABAS achieved the best performance, then the ResUNet, and DeepLabv3+ ranked the last based on the volumetric DSC (mean for the right femoral head, ABAS *vs*. ResUNet *vs*. DeepLabv3+, 0.94 *vs*. 0.85 *vs*. 0.84, P< 0.01) and surface DSC (mean for the right femoral head, ABAS *vs*. ResUNet *vs*. DeepLabv3+, 0.84 *vs*. 0.70 *vs*. 0.67, P< 0.01), despite more outliers in volumetric DSC of ABAS. The ground truth and the automatic delineation of a random case were shown in **Figure 4**.

The D_Abs_ of the dose parameters between Plan_test_ and Plan_ref_ were shown in **Table 3**, and it contained descriptive statistics and results of statistical tests. We observed a statistical difference in dose distribution of real CTV between the Plan_ref_ and Plan 1-3 (P< 0.01), which used automatically delineated CTV as an inverse optimization parameter. The difference, however, was that some dose parameters of real OAR in Plan1 were not significantly different from the Plan_ref_ (P > 0.05), which showed a similar trend to the outperformance of DeepLabv3+ in geometric evaluation. When we introduced the auto-segmentation OAR groups into the inverse plan, we found that only critical dose parameters of the small intestine between Plan5 and the Plan_ref_ had no statistical difference (P > 0.05), but the small intestine generated by ResUNet was not optimal in the geometric assessment (volumetric DSC, mean = 0.84; surface DSC, mean = 0.52).

**Table 3:** Statistical analysis of dose parameter difference between test and reference plans.

ROI	Dose Parameter	Plan1 (CTV_DeepLabv3+_ and OAR_GT_)		Plan2 (CTV_ResUNet_ and OAR_GT_)		Plan3 (CTV_ABASt_ and OAR_GT_)		Plan4 (OAR_DeepLabv3+_ and CTV_GT_)		Plan5 (OAR_ResUNet_ and CTV_GT_)		Plan6 (OAR_ABAS_ and CTV_GT_)	
		P^a^	D_Abs_^b^	P	D_Abs_	P	D_Abs_	P	D_Abs_	P	D_Abs_	P	D_Abs_
CTV_GT_	V50.4	<0.01	3.74(4.34)	<0.01*	4.14(5.27)	<0.01	6.39(9.31)	0.04	1.87(2.80)	**0.24**	1.61(2.23)	<0.01	2.17(3.95)
PTV_GT_	D2	<0.01*	0.40(0.30)	<0.01	0.34(0.35)	<0.01	0.61(0.42)	<0.01	0.23(0.19)	0.02	0.19(0.35)	0.03	0.32(0.42)
	D95	<0.01*	4.15(4.20)	<0.01*	6.82(4.66)	<0.01	20.36(80.90)	<0.01	0.15(0.19)	**0.12**	0.13(0.17)	0.04	0.32(0.46)
	D98	<0.01*	10.43(7.60)	<0.01	12.71(7.93)	<0.01	41.28(77.17)	<0.01	0.19(0.25)	0.02	0.18(0.22)	**0.16**	0.44(0.68)
	CI	<0.01	0.11(0.04)	<0.01	0.13(0.05)	<0.01	0.20(0.08)	0.03	0.01(0.02)	**0.23**	0.01(0.02)	**0.08**	0.03(0.05)
	HI	<0.01*	0.10(0.08)	<0.01*	0.12(0.08)	<0.01	0.40(0.76)	<0.01	0.003(0.004)	<0.01	0.003(0.003)	<0.01	0.01(0.01)
Bladder_GT_	D2	<0.01	0.40(0.61)	0.01	0.40(0.53)	<0.01	0.44(0.61)	0.11*	0.19(0.39)	0.14	0.29(0.40)	**0.83**	0.36(0.48)
	V30	**0.72**	5.34(5.29)	**0.11**	5.78(6.55)	<0.01	18.49(10.37)	0.01*	2.21(3.07)	0.01*	2.56(4.14)	<0.01	23.89(9.01)
	V40	**0.28**	4.58(7.34)	0.01	6.01(8.22)	<0.01*	16.71(13.28)	0.36*	1.59(2.07)	0.35*	1.53(1.91)	<0.01	22.87(12.91)
	V50	**0.12**	5.67(6.65)	0.01	5.84(5.65)	0.09*	8.04(7.19)	<0.01*	0.90(1.68)	<0.01*	1.11(1.31)	**0.06**	1.11(2.39)
Small intestine_GT_	D2	0.16*	0.32(0.37)	0.01*	0.29(0.42)	<0.01	0.47(0.76)	**0.82**	0.11(0.19)	**0.49**	0.18(0.17)	**0.14**	0.26(0.49)
	V15	<0.01*	3.10(3.04)	<0.01*	3.44(3.60)	0.04	8.63(17.07)	0.20*	1.04(1.84)	0.56*	1.08(1.82)	<0.01	3.83(7.82)
	V45	0.02	2.06(3.42)	<0.01	2.64(3.68)	0.97*	3.30(5.35)	0.02*	0.26(0.66)	0.13*	0.24(0.60)	<0.01*	4.09(4.33)
	V50	0.04	1.92(2.57)	<0.01	1.92(3.44)	<0.01*	2.05(3.84)	0.12*	0.24(0.30)	0.54*	0.18(0.37)	**0.95**	0.26(0.67)
Right Femoral Head_GT_	D2	**0.17**	4.75(7.34)	**0.06**	4.14(5.28)	<0.01*	5.29(4.68)	<0.01	9.40(8.06)	<0.01	9.12(10.53)	0.81*	4.42(5.65)
	V40	0.12*	0.38(1.15)	0.01*	0.21(0.46)	<0.01	3.43(2.41)	<0.01*	0.99(2.02)	<0.01*	1.16(2.95)	<0.01	5.80(5.44)
	V45	0.10*	0.03(0.25)	0.03*	0.002(0.05)	0.05	0.03(0.19)	<0.01*	0.05(0.39)	<0.01*	0.08(0.35)	<0.01	0.32(1.18)
Left Femoral Head_GT_	D2	**0.71**	4.95(6.22)	<0.01	5.00(5.82)	<0.01*	10.07(8.78)	<0.01	8.14(9.27)	<0.01	8.11(7.12)	0.20*	5.19(7.16)
	V40	0.97*	0.63(1.17)	<0.01	0.85(1.23)	<0.01	2.63(2.56)	<0.01*	1.94(2.89)	<0.01	1.86(2.58)	<0.01*	7.17(7.15)
	V45	0.98*	0.13(0.54)	<0.01*	0.09(0.39)	**0.13**	0.14(0.40)	<0.01*	0.36(0.73)	<0.01*	0.44(0.74)	<0.01	1.11(1.72)

a. P values (2-tailed) marked with ^*^ indicated results of paired samples Wilcoxon signed rank test for original dose parameters, while the unmarked P values were the results of paired samples t tests.

b. D_Abs_ were the absolute difference between specific dose parameters extracted from the test plans and the reference plan, described by the median (IQR). In particular, the difference of Dn was normalized according to the δDn formula. P values with no statistical significance were bold.

Although the volumetric and surface DSCs of all structures were numerically different, there was a correlation between them (P< 0.01). The correlation analysis results of geometric metrics and dose parameters were shown in **Table 4**. The volumetric and surface DSCs of CTV generated by ResUNet were correlated with all dose parameters in target volume (P< 0.05) in Plan2, on the other hand, the volumetric DSC of the three CTV groups were correlated with δD95, δD98, HI, and CI (P< 0.05) respectively in Plan 1-3 . For the bladder, the volumetric DSC of ResUNet results was correlated with all dose parameters of the bladder in Plan5, and δD2 in Plan 4-6 was correlated with both DSC metrics. There were few correlation indexes in the small intestine, only volumetric DSC *vs*. V15 and surface DSC *vs*. V45/V50. In the results of bilateral femoral heads, both DSC metrics of two CNNs were correlated with the corresponding δD2 in Plan 4-5.

**Table 4:** Pearson correlation analysis between geometric parameters and corresponding multiple dose parameters of the same structure.

		Volumetric DSC			Surface DSC		
		DeepLabv3+	ResUNet	ABAS	DeepLabv3+	ResUNet	ABAS
CTV	δD2	-0.31^*^	-0.34^*^	0.11	-0.28	-0.38^**^	0.01
	δD95	-0.54^**^	-0.79^**^	-0.79^**^	-0.41^**^	-0.66^**^	-0.15
	δD98	-0.43^**^	-0.68^**^	-0.77^**^	-0.31^*^	-0.57^**^	-0.19
	HI	-0.39^**^	-0.67^**^	-0.77^**^	-0.28	-0.57^**^	-0.19
	CI	-0.31^*^	-0.57^**^	-0.45^**^	-0.30^*^	-0.60^**^	-0.56^**^
Bladder	V30	-0.27	-0.30^*^	-0.41^**^	-0.24	-0.28	-0.55^**^
	V40	-0.28	-0.34^*^	-0.25	-0.26	-0.31^*^	-0.38^*^
	V50	-0.23	-0.34^*^	0.25	-0.20	-0.27	0.22
	δD2	-0.37^*^	-0.46^**^	-0.31^*^	-0.36^*^	-0.41^**^	-0.42^**^
Small Intestine	V15	0.20	0.17	0.50^**^	0.01	<0.01	-0.08
	V45	-0.05	-0.11	0.14	-0.16	-0.20	-0.37^*^
	V50	-0.06	-0.09	0.11	-0.15	-0.16	-0.39^*^
	δD2	0.23	<-0.01	-0.06	0.19	-0.11	-0.02
Right Femoral Head	V40	-0.21	-0.25	0.21	-0.21	-0.26	0.18
	V45	-0.14	-0.14	-0.02	-0.14	-0.13	-0.02
	δD2	-0.45^**^	-0.51^**^	-0.42^**^	-0.46^**^	-0.52^**^	-0.30
Left Femoral Head	V40	-0.28	-0.20	0.39^**^	-0.31^*^	-0.22	0.44^**^
	V45	-0.17	-0.11	0.16	-0.19	-0.12	0.28
	δD2	-0.42^**^	-0.38^**^	0.07	-0.43^**^	-0.40^**^	0.08

The values in the table were Pearson correlation coefficient “r”, r values marked with ^**^ indicated a significant correlation at test level 0.01 (2-tailed), r values marked with ^*^ indicated a significant correlation at test level 0.05 (2-tailed), and unmarked r values indicated no significant correlation. PTV was generated by CTV plus a uniform margin, so we tested the dose parameters of PTV with the geometric parameters of CTV.

## 4 Discussion

In our retrospective study, geometric and dosimetric evaluations of CTV and OARs for rectal cancer were carried out using manually segmented structures as the ground truth, while commercial software ABAS generated structures as reference. The results showed that the CNN models had a remarkable performance in accuracy and repeatability of automatic segmentation, but their performance in geometric metrics and dose parameters was not completely consistent.

The effect of automatic segmentation requires objective metrics for evaluation. Volumetric DSC is the most commonly used metric and describes the degree of overlap between two structures; however, it weights all misplaced segmentations equally and cannot characterize the distance of the ROI surface. For example, a structure with more proportion needs to be modified slightly and takes a long time may obtain a high volumetric DSC, while a structure requiring a large amount of modification locally and a short time-consuming may have a low volumetric DSC. For the description of surface distance, a commonly used metric is the HD, which represents the maximum of the shortest distances from any delineated point to the other contour (23). At the same time, the limitation lies in its description of the maximum surface distance rather than the degree of surface difference of the entire structure. Therefore, our study includes the surface DSC. This metric has the function of subjective and objective assessment, which contains tolerable interobserver subjective errors, describes the degree of overlap from the perspective of the entire structure, and to a certain extent characterizes the potential cost of modification. Some studies have counted the time spent on performing manually correcting auto-contouring (24). In our previous study, we calculated the computing time of CNNs, in which the average time for generating a case was about 28 s for DeepLabv3+ and 35 s for ResUNet; and we recorded the manual correction time of the results, in which the average cost for CTV_DeepLabv3+_, CTV_ResUNet_, and all OARs was about 11 min, 7 min, and below 5 min respectively (13). With large inter-observer variability, it can still be determined that the manual contouring time is much greater than the total time of automatic contouring and manual modification.

Besides geometric accuracy, the effect of automatically generated ROIs on treatment planning should also be considered. Since the dose distribution is affected by mechanical and physical factors and cannot fully fit the edge of the structure, parts of the automatic contouring that are not perfectly consistent with clinical ground truth may be covered by the isodose lines, which perhaps can be considered as the “robustness” of the structure. Therefore, the evaluation of automatic delineation results should be combined with dosimetry results rather than a single geometric evaluation. There are many methods for dose evaluation; the simplest is to transplant a reference plan into different ROI sets and calculate the dose parameters for comparison (15). However, these parameters do not exist dose distribution replanned using different optimization objects is hardly consistent with the reference plan. Another method is to generate a plan for each group of ROIs and compare their dose characteristics without any reference. We consult the evaluation method for interobserver variation, which assesses plans generated from different ROI sets by calculating the dose parameters of the reference structures (25). Based on this method, we add the definition of “ground truth”, design plans using CTV and OAR groups separately and carry out dosimetry evaluation by ROI_GT_. The results indicate that some crucial dose parameters in the actual plan have no significant difference from the reference plan even if the structure of automatic segmentation cannot completely overlap with ROI_GT_, especially in the automatically delineated OARs. Interestingly, it can be seen from Plan1 that DeepLabv3+ has a smaller effect on OAR dose distribution, following a similar trend to its dominance in DSC metrics. However, ResUNet-generated small intestine did not show an advantage in the geometric assessment, but its dose parameters in Plan5 did not differ from those in Plan_ref_. Therefore, we cannot only pursue the improvement of geometric metrics, perhaps the performance assessment of auto-segmentation methods should be combined with dose evaluation, as the latter is more relevant to clinical outcome (26).

At present, our institution has established an integrated platform for automatic delineation (including head, chest, abdomen, pelvis, etc.), which is connected to TPS and CT workstations through the hospital’s internal network. The platform can realize CT image transmission, conversion between RS files and masks, and continuous input of abnormal cases (such as recognizing the skull as the femoral head) to improve network performance. This is also a key step in the real application of artificial intelligence to the clinic. The auto-segmentation assessment should integrate subjective and objective methods, but the subjective assessment will introduce inter-observer variability, so it needs multi-center external validation (27). This is a limitation of this study, and we plan to extend the integrated platform to other hospitals and collect data for external validation. In addition, the dose evaluation method also needs to be further improved. In this study, all structures are divided into CTV and OAR groups, and the results may be more targeted and reliable if each structure is taken as a separate variable of the optimization object. In terms of analysis methods, the paired test of dose parameters was used in this study; and what if directly compare RT Dose files and assess dose difference from both global and local perspectives.

## 5 Conclusion

In this study, we evaluated the automatic segmentation results from the perspectives of geometry and dosimetry. The results showed the advantages of speed and repeatability of deep learning in ROI delineation, which is of great help to the routine workflow of radiotherapy. The auto-segmentation function of CNNs is a stability tool for VMAT and IMRT treatment plan design, and it may have further potential in adaptive radiotherapy, which requires repeat CT scans and CTV delineation before each treatment fraction (28). Moreover, with the advancement of MR-Linac, automatic segmentation based on magnetic resonance images has been applied to adaptive radiotherapy, which poses a great challenge to the speed, accuracy, and effect on the dose distribution of the networks (29, 30).

The characteristics of convolutional neural networks are different, and the segmentation effect on the ROIs of rectal cancer also differs. We can integrate the two networks or classify them according to the advantageous structure of each network; however, whether further exploration can bring better results requires a comprehensive clinical evaluation.

## Data availability statment

The original contributions presented in the study are included in the article. Further inquiries can be directed to the corresponding author.

## Ethics statement

The studies involving humans were approved by Ethics Committee on Biomedical Research, West China Hospital of Sichuan University. The studies were conducted in accordance with the local legislation and institutional requirements. Written informed consent for participation was not required from the participants or the participants’ legal guardians/next of kin in accordance with the national legislation and institutional requirements.

## Author contributions

JL, YS, and SB contributed to conception and design of the study. SB and GL contributed to administrative support and provision of study materials. YS organized the database. JL, YW, and LL collected the data. JL performed the statistical analysis. All authors contributed to the manuscript and approved the submitted version.
